# Determinants of Healthcare-Associated Infections in King Abdulaziz Specialized Hospital in Taif, Saudi Arabia

**DOI:** 10.7759/cureus.69423

**Published:** 2024-09-14

**Authors:** Malak Aloufi, Mohammed E Aloufi, Shrouq R Almalki, Noha Saleh M Hassanien

**Affiliations:** 1 Preventive Medicine, Health Cluster, Taif, SAU; 2 Public Health, Health Cluster, Taif, SAU; 3 Biostatistics, High Institute of Public Health, Alexandria University, Alexandria, EGY

**Keywords:** bloodstream infections (bsi), catheter-associated urinary tract infections (cauti), central line-associated bloodstream infections (clabsi), comorbidity, determinants, fever, healthcare-associated infections

## Abstract

Background: Healthcare-associated infections (HAIs) represent a significant challenge in hospital settings, contributing to increased morbidity, mortality, and healthcare costs. This study aimed to estimate the prevalence and socio-demographic and clinical determinants of HAIs at the King Abdulaziz Specialized Hospital (KAASH) in Taif, Saudi Arabia.

Methodology: A hospital-based cross-sectional study was conducted from March 2023 to January 2024 targeting inpatients aged 18 and above in all units and wards. Data were collected using the National Healthcare Safety Network (NHSN) criteria for definitions of surveillance. A structured questionnaire gathered socio-demographic and clinical data from patients or next of kin if the patient was not fully oriented. Descriptive statistics were performed, and analytical methods used included Pearson chi-square test, Pearson correlation, independent t-test, and one-way analysis of variance.

Results: Among 318 participants included in this study, the mean age of participants was 56.44 years, with a slight female predominance (n=164, 51.6%). Hypertension (n=162, 50.9%) and diabetes (n=126, 39.6%) were the most prevalent comorbidities. Pneumonia (n=60, 26.8%) and trauma (n=55, 17.4%) were the leading causes of admission. The two most common HAIs included catheter-associated urinary tract infections (CAUTI) (n=124, 39%) and central line-associated bloodstream infections (CLABSI) (n=74, 23.3%). The primary causative organisms were *Klebsiella pneumoniae *(n=96, 30.2%) and *Acinetobacter baumannii* (n=32, 10.1%). The most significant predictors of HAIs were as follows: For CLABSI, risk factors include having three or more comorbidities, fever above 37.8°C, chills or rigors, hypotension, and positive blood culture. For CAUTI, key predictors were urinary tract infection (UTI), positive urine culture, acute pain or swelling of the testes, suprapubic tenderness, visible hematuria, and leukocytosis. Significant predictors of bloodstream infections (BSI) include having a BSI, positive blood culture, chills or rigors, and hypotension. Fever and hypotension increased CLABSI and BSI risk but reduced the CAUTI risk.

Conclusion: The study highlights a significant burden of HAIs at the KAASH, with multiple predictors. The findings underscore the need for robust infection control measures and targeted interventions to reduce HAI incidence and improve patient outcomes.

## Introduction

Healthcare-associated infections (HAIs) are infections that develop in patients when they are receiving treatment for their medical or surgical conditions. They are considered a serious problem in healthcare services as they are common causes of new illnesses that the patient did not have before entering the hospital [1]. HAIs are associated with a considerable increase in morbidity rates, mortality, length of stay, disability, and increasing healthcare costs [2-4]. HAI is a major concern for increased resistance of microorganisms due to the use of antibiotics to treat such conditions [5]. Point-prevalence surveys of HAIs in healthcare settings conducted showed that overall point prevalence was 6.8% (114 of 1,666). The most common types of infections were pneumonia (27.2%), urinary tract infections (UTIs) (20.2%), and bloodstream infections (BSI) (10.5%), while 19.2% of HAIs were device-associated [6]. Studies have shown that HAIs are primarily associated with microbial resistance, inadequate infection control practices, patient environment, staff training, and surveillance [7,8].

A study in the Middle East highlighted that infrastructure-related factors, such as hospital overcrowding and inadequate patient spacing, also contribute to infection rates [7]. HAIs at King Abdulaziz Specialist Hospital (KAASH) in Taif, Saudi Arabia, have been a significant concern, particularly in the neonatal intensive care unit (NICU). A study conducted in 2013 highlighted a prevalence (6.03%), with a death rate of 27.1% of HAIs at KAASH, with Klebsiella species identified as a major pathogen responsible for infections in neonates [9]. This is similar to the findings in the neighboring countries (Jordan, Kuwait, Lebanon, and Qatar) and globally, where antibiotic-resistant bacteria, such as Klebsiella spp., are prevalent in the NICU, leading to increased morbidity and mortality [7,10,11]. This situation reflects broader trends observed in healthcare settings across the region, where inadequate infection control measures and delayed recognition of outbreaks have contributed to the persistence of HAIs [7]. However, no recent studies have explored this disease burden at KAASH to inform updated measures to ensure the quality of care and optimize patient outcomes. This gap limits the ability of healthcare professionals and policymakers to implement targeted interventions that could mitigate the impact of HAIs and improve patient outcomes. Therefore, this study aimed to estimate the prevalence and determinants of HAIs at KAASH in Taif, Saudi Arabia.

The rationale for this study is grounded in the urgent need to update our understanding of the prevalence and determinants of HAIs at KAASH. Previous research conducted at KAASH identified a significant prevalence of HAIs and a concerning mortality rate, particularly [9]. However, this research is now outdated, and the healthcare landscape has evolved, necessitating a re-evaluation of the situation. In addition, HAIs are closely associated with antimicrobial resistance (AMR), a growing global health crisis, and the prevalence of antibiotic-resistant bacteria, such as Klebsiella species, particularly in the NICU setting, underscores the critical need for comprehensive infection control measures that address both prevention and the prudent use of antibiotics [7,10]. Understanding the current prevalence and determinants of HAIs at KAASH will provide essential insights into the relationship between infection control practices, microbial resistance, and patient outcomes, facilitating the development of tailored interventions.

This study is justified by the pressing need for updated data to guide the enhancement of infection control protocols at KAASH. The findings will not only help address the specific challenges faced by this hospital but will also contribute to the broader efforts to combat HAIs and AMR in healthcare settings across the region. By identifying the determinants of HAIs, this research will inform strategies to optimize patient safety, improve healthcare outcomes, and reduce the economic burden associated with HAIs. Furthermore, this study will fill a critical knowledge gap in the literature, providing a foundation for future research and policy development aimed at reducing the impact of HAIs in Saudi Arabia and similar healthcare environments.

## Materials and methods

Study setting and design

This study was a cross-sectional study conducted at the KAASH in Taif, Saudi Arabia, targeting inpatients aged 18 and above in all units and wards of the hospital.

Participants

This study enrolled patients who showed no evidence of bacterial infections upon admission. Patients aged 18 and above admitted at the KAASH from March 2023 to January 2024 in wards (Intensive Care Unit (ICU), Coronary Care Unit (CCU), High Dependency Unit (HDU), medical ward, surgical ward, psychiatric ward, and oncology) were included. Exclusion criteria encompassed patients under 18, those in outpatient services, emergency departments, skilled nursing, and rehabilitation units, as well as patients who developed infections within 48 hours of admission or had asymptomatic bacteriuria.

Data collection

Data collection involved surveys of inpatients using the National Healthcare Safety Network (NHSN) criteria for surveillance definitions [12]. Socio-demographic and clinical data were gathered using a structured questionnaire. The questionnaire collected information on age, gender, residency, occupation, education, marital status, disease admitted with, type of HAIs developed, symptoms, body mass index (BMI), investigation results, defining organism, and possible cause and entry of the infected agent. All data were registered on a case report form (CRF). A convenience sampling technique was used, and all eligible patients available during the study period were enrolled.

Variables and outcomes

The dependent variable in this study was the development of HAIs, with independent variables including length of stay and other co-morbidities. The primary outcome variable was defined as the occurrence of infection after 48 hours of hospitalization in patients who did not have symptomatic or incubating infections upon admission. HAIs were categorized according to the CDC/NHSN categories and included criteria for BSI, healthcare-associated pneumonia, surgical site infections (SSI), and UTIs.

Statistical analysis 

Data were analyzed using the Statistical Program for Social Sciences (SPSS) version 25 (IBM, Armonk, NY, USA). Descriptive statistics were calculated, and analytical methods included Pearson chi-square test, independent t-test, and one-way analysis of variance (ANOVA) test at a 95% CI. Logistic regression analyses were performed, odds ratios (OR) were calculated, and CIs were calculated to determine the strengths of associations. A statistical significance was considered at p-value <0.05.

Ethical considerations

We obtained approval from the Saudi Ministry of Health Directorate of Health Affairs Institutional Review Board (IRB), Taif, Saudi Arabia (Ref. No: 780), higher authority, local or institutional, and departmental levels. Participants were informed of the study's purpose, assured of confidentiality, and informed of their right to withdraw at any time without obligation. Written consent was obtained from participants or their closest relatives if the patients were comatose.

## Results

The study sample (N=318) had a mean age of 56.44 years (SD=22.32), with a roughly equal gender distribution (51.6% female, 48.4% male). The majority of participants resided in Taif (n=242, 76.1%) and were married (n=206, 64.8%). Occupationally, the largest group was engaged in their own businesses (n=132, 41.5%), followed by housewives (n=60, 18.9%) and retirees (n=56, 17.6%). Notably, 256 (80.5%) of participants reported at least one comorbidity, with 100 (31.4%) having two and 82 (25.8%) having three or more. Most participants were non-smokers (n=242, 76.1%), and 148 (46.5%) had normal weight. A significant proportion (n=192, 60.4%) were bedridden (Table 1).

**Table 1:** Demographic and clinical characteristics of the participants BMI, body mass index

		Count	Column N%
Age	Mean (SD)	56.44 (22.32)	
Gender	Female	164	51.6%
	Male	154	48.4%
Residency	Taif	242	76.1%
	Outside Taif	76	23.9%
Occupation and education	Housewife	60	18.9%
	Student	42	13.2%
	Employee	22	6.9%
	Military	6	1.9%
	Retired	56	17.6%
	Own business	132	41.5%
Marital state	Single	112	35.2%
	Married	206	64.8%
Comorbidities	No	62	19.5%
	One	74	23.3%
	2 comorbidities	100	31.4%
	3 or more	82	25.8%
Smoker	No	242	76.1%
	Former smoker	24	7.5%
	Active smoker	52	16.4%
BMI	Under	84	26.4%
	Normal	148	46.5%
	High	86	27.0%
Nonsurgical wound or injury	No	222	69.8%
	Yes, clean	80	25.2%
	Yes, contaminated	16	5.0%
Physical mobility	Without assistance	42	13.2%
	With assistance	84	26.4%
	Bedridden	192	60.4%

**Figure 1:**
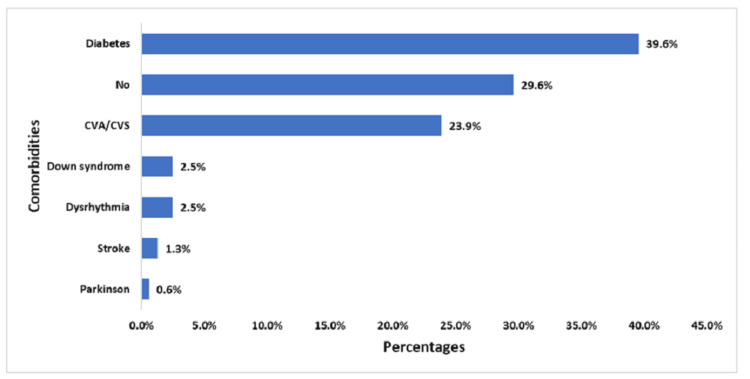
Comorbidities among the participants

The study revealed diverse comorbidities and reasons for hospital admission among the 318 participants. Diabetes emerges as the most prevalent comorbidity, affecting 39.6% of the population. Cardiovascular conditions represented by CVA/CVS were the second most common specific comorbidity at 23.9%. Neurological and developmental disorders are also present, albeit at lower rates: Down syndrome and dysrhythmia, each at 2.5%, stroke at 1.3%, and Parkinson's disease at 0.6%. Notably, 29.6% of comorbidities had no comorbidities (Figure 1).

**Figure 2:**
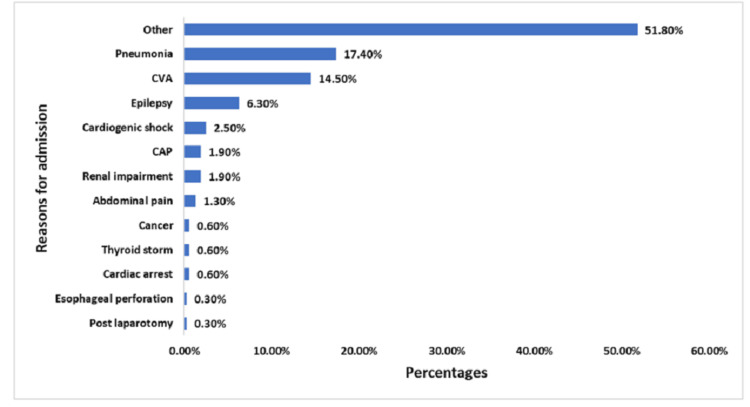
Reasons for hospital admission among participants

In addition, the results revealed a diverse range of admission reasons, with "Other" causes accounting for the majority (51.80%) of admissions. Pneumonia emerges as the second most common specific reason for admission at 17.40%. Cerebrovascular accidents (CVA) follow closely at 14.50%. Neurological conditions are also prominent, with epilepsy accounting for 6.30% of admissions. Cardiovascular issues such as cardiogenic shock (2.50%) and cardiac arrest (0.60%) collectively contribute to a noteworthy proportion of admissions. Other reasons for admission include respiratory (CAP, likely community-acquired pneumonia, at 1.90%), renal (1.90%), and gastrointestinal conditions (abdominal pain at 1.30%, esophageal perforation at 0.30%). Endocrine disorders (thyroid storm) and oncological issues (cancer) each account for 0.60% of admissions (n=20, 6.3%) (Figure 2).

A study of 318 participants found that 33.3% (n=106) were admitted to the High Dependency Unit (HDU), 30.8% (n=98) to surgical wards, 16.4% (n=54) to the Intensive Care Unit (ICU), and 11.3% (n=36) to medical wards. The most common HAI was catheter-associated urinary tract infection (CAUTI), affecting 39% (n=124) of patients, followed by central line-associated bloodstream infections (CLABSI) (n=74, 23.3%), and BSI (n=70, 22%). The occurrence of HAIs varied by ward, with the highest incidence in the HDU (n=110, 34.6%) and surgery wards (n=98, 30.8%). *Klebsiella pneumoniae* was the most common pathogen, causing 30.0% (n=98) of cases, followed by *Acinetobacter baumannii* and *Escherichia coli*, each causing 10.1% (n=32) of cases (Table 2). 

**Table 2:** Results of hospital-acquired infections and admission characteristics HAIs, healthcare-associated infections; HDU, High Dependency Unit; ICU, Intensive Care Unit; CCU, Coronary Care Unit; CAUTI, catheter-associated urinary tract infections; CLABSI, central line-associated bloodstream infections; BSI, bloodstream infections; ABUTI, asymptomatic bacteremic urinary tract infection;* *SSI, surgical site infections

		Count	Column N%
Admission ward	Female medical	2	0.6%
	HDU	106	33.3%
	Oncology	12	3.8%
	Surgery	98	30.8%
	Medical	36	11.3%
	CCU	10	3.1%
	PSYCH	2	0.6%
	ICU	52	16.4%
Type of HAIs developed	CLABSI	74	23.3%
	CAUTI	124	39.0%
	BSI	70	22.0%
	SSI	18	5.7%
	ABUTI	6	1.9%
	PVAP	12	3.8%
	DECU	14	4.4%
HAI ward	ICU	52	16.4%
	HDU	110	34.6%
	Oncology	10	3.1%
	Surgical	98	30.8%
	Medical	36	11.3%
	CCU	10	3.1%
	PSYCH	2	0.6%
Organism	Klebsiella pneumoniae	96	30.0%
	Proteus mirabilis	12	3.8%
	Acinetobacter baumannii	32	10.1%
	Candida dublinensis	16	5.0%
	MRSA	16	5.0%
	Serratia marcescens	8	2.5%
	Pseudomonas aeruginosa	20	6.3%
	Enterococcus faecium	16	5.0%
	Enterobacter cloacae	11	3.5%
	Providencia staurtii	2	0.6%
	Stenotrophomonas maltophilia	5	1.6%
	Staphylococcus aureus	11	3.5%
	Escherichia coli	32	10.1%
	Enterobacter aerogenes	5	1.6%
	Candida species	14	4.4%
	Acinetobacter baumanii	18	5.7%
	Klebsiella oxytoca	4	1.3%

The study analyzed the relationship between three common HAIs (CLABSI, CAUTI, and BSI) and demographic factors. Both CAUTI (p<0.001) and BSI (p<0.001) were significantly associated with invasive procedures. For CLABSI, female participants were more significantly affected (p=0.009). A significant relationship was found between nonsurgical wounds or injuries and BSI, with those having contaminated wounds showing a lower incidence (P=0.026). Moreover, the presence of comorbidities showed a statistically significant relationship (P=0.025), with those with three or more comorbidities showing the highest incidence. A significant relationship was found between nonsurgical wounds or injuries and CAUTI, with those having contaminated wounds showing the lowest incidence (P=0.007). Comorbidities were significant, with the highest CAUTI rate (n=116, 70.7%) among those with three or more comorbidities (P=0.045) (Table 3).

**Table 3:** The relation between the three most common HAIs and demographic factors *Significant p-value <0.05 CLABSI, central line-associated bloodstream infections; CAUTI, catheter-associated urinary tract infections; BSI, bloodstream infections; BMI, body mass index

		CLABSI					CAUTI					BSI				
		No		Yes		P-value	No		Yes		P-value	No		Yes		P-value
		Count	Row N %	Count	Row N %	P-value	Count	Row N %	Count	Row N %	P-value	Count	Row N %	Count	Row N %	P-value
Age	Mean (SD)	54.59 (22.45)		62.54 (20.87)		0.007*	58.04 (23.68)		53.94 (19.85)		0.11	56.67 (20.47)		54.91 (28.02)		0.518
Gender	Male	128	83.10%	26	16.90%	0.009*	98	63.60%	56	36.40%	0.351	110	71.40%	44	28.60%	0.006*
Gender	Female	116	70.70%	48	29.30%		96	58.50%	69	41.50%		138	84.10%	26	15.90%	
Residency	Outside Taif	64	84.20%	12	15.80%	0.077	52	68.40%	24	31.60%	0.129	56	73.70%	20	26.30%	0.299
Residency	Taif	180	74.40%	62	25.60%	0.077	142	58.70%	100	41.30%	0.129	192	79.30%	50	20.70%	
Occupation and education	Housewife	44	73.30%	16	26.70%	0.192	36	60.00%	24	40.00%	0.957	48	80.00%	12	20.00%	0.143
Occupation and education	student	36	85.70%	6	14.30%		28	66.70%	14	33.30%		26	61.90%	16	38.10%	
Occupation and education	Employee	14	63.60%	8	36.40%		12	54.50%	10	45.50%		18	81.80%	4	18.20%	
Occupation and education	Military	6	100.00%	0	0.00%		4	66.70%	2	33.30%		4	66.70%	2	33.30%	
Occupation and education	Retired	40	71.40%	16	28.60%		34	60.70%	22	39.30%		44	78.60%	12	21.40%	
Occupation and education	Own business	104	78.80%	28	21.20%		80	60.60%	52	39.40%		108	81.80%	24	18.20%	
Marital state	Single	89	80.20%	22	19.80%	0.286	69	62.20%	42	37.80%	0.757	83	74.80%	28	25.20%	0.311
Marital state	Married	155	74.90%	52	25.10%		128	60.40%	82	39.60%		165	79.70%	42	20.40%	
Smoker	No	186	76.90%	56	23.10%	0.399	142	58.70%	100	41.30%	0.299	192	79.30%	50	20.70%	0.573
Smoker	Former smoker	16	66.70%	8	33.30%		18	75.00%	6	25.00%		18	75.00%	6	25.00%	
Smoker	Active smoker	42	80.80%	10	19.20%		34	65.40%	18	34.60%		38	73.10%	14	26.90%	
Comorbidities	No	54	87.10%	8	12.90%	0.025*	38	61.30%	24	38.70%	0.045*	46	74.20%	16	25.80%	0.59
Comorbidities	One	58	78.40%	16	21.60%		36	48.60%	38	51.40%		56	75.70%	18	24.30%	
Comorbidities	2 comorbidities	78	78.00%	22	22.00%		62	62.00%	38	38.00%		78	78.00%	22	22.00%	
Comorbidities	3 or more	54	65.90%	28	34.10%		58	70.70%	24	29.30%		68	82.90%	14	17.10%	
BMI	Under	64	76.20%	20	23.80%	0.990	60	71.40%	24	28.60%	0.07	58	69.00%	26	31.00%	0.055
BMI	Normal	114	77.00%	34	23.00%		86	58.10%	62	41.90%		118	79.70%	30	20.30%	
BMI	High	66	76.70%	20	23.30%		48	55.80%	38	44.20%		72	83.70%	14	16.30%	
Nonsurgical wound or injury	No	166	0.748	56	0.252	0.069	124	55.90%	98	44.10%	0.007*	182	82.00%	40	18.00%	0.026*
Nonsurgical wound or injury	Yes, clean	62	77.50%	18	22.50%		56	70.00%	24	30.00%		54	67.50%	26	32.50%	
Nonsurgical wound or injury	Yes, contaminated	16	100.00%	0	0.00%		14	87.50%	2	12.50%		12	75.00%	4	25.00%	
Physical mobility	Without assistance	34	81.00%	8	19.00%	0.659	24	57.10%	18	42.90%	0.520	32	76.20%	10	23.80%	0.135
Physical mobility	With assistance	62	73.80%	22	26.20%		48	57.10%	36	42.90%		72	85.70%	12	14.30%	
Physical mobility	Bedridden	148	77.10%	44	22.90%		122	63.50%	70	36.50%		144	75.00%	48	25.00%	
Length of hospitalization	Mean (SD)	91.85 (144.14)		61.86 (62.50)		0.083	56.90 (78.67)		128.6 (175.8)		<0.001*	92.49 (136.2)		57.89 (103.1)		0.05*
Prior pharmacological and/or non-pharmacological therapy	No	54	87.10%	8	12.90%	0.023*	32	51.60%	30	48.40%	0.15	46	74.20%	16	25.80%	0.189
Prior pharmacological and/or non-pharmacological therapy	Yes	176	73.30%	64	26.70%	0.023*	148	61.70%	92	38.30%	0.15	196	81.70%	44	18.30%	0.189
Invasive procedures	IV line	170	78.00%	48	22.00%	0.737	148	67.90%	70	32.10%	<0.001*	154	70.60%	64	29.40%	<0.001*
Invasive procedures	Mechanical ventilator	20	71.40%	8	28.60%	0.737	20	71.40%	8	28.60%	<0.001*	24	85.70%	4	14.30%	<0.001*
Invasive procedures	Urinary catheter	50	75.80%	16	24.20%	0.737	22	33.30%	44	66.70%	<0.001*	64	97.00%	2	3.00%	<0.001*

The study analyzed the clinical symptoms and factors associated with HAIs among 318 participants. Fever was present in 58.9% (n=186) of cases, while UTIs were identified in 40.9% (n=130) of patients. Suprapubic tenderness was noted at 13.9% (n=44), and visible hematuria at 8.8% (n=28). Leukocytosis was found in 34.6% (n=110) of cases, with 30.8% (n=98) experiencing chills or rigor and 28.3% (n=90) suffering from hypotension. A significant majority of patients (n=240, 79.5%) had prior pharmacological and/or nonpharmacological therapy, and 63.3% (n=200) received preoperative prophylaxis. Fever (>37.8°C) is significantly associated with CLABSI (P<0.001) and CAUTI (P = 0.006). UTIs are highly associated with CAUTI (P<0.001). Positive urine culture is also significantly linked to CAUTI (P<0.001). Acute dysuria (9.7%, P = 0.002), acute pain/swelling/tenderness of the testes, epididymis, or prostate (P<0.001), costovertebral angle pain (P<0.001), suprapubic tenderness (P<0.001), and visible hematuria (P<0.001) are all significantly associated with CAUTI. Leukocytosis is significant for CLABSI (P<0.001) and CAUTI (P<0.001). BSI (P<0.001), chills/rigors (P<0.001), and hypotension (P<0.001) were also significantly associated with CLABSI. At least one positive blood culture was significantly associated with CLABSI (P<0.001) and BSI (P<0.001). Spreading cellulitis (P<0.001), purulent drainage (P=0.002), surgical site infections (P=0.005), and abscess (P=0.035) were significantly associated with CLABSI, and prior pharmacological therapy was notable for CLABSI (P=0.023) (Table 4).

**Table 4:** Clinical symptoms and associated factors in hospital-acquired infections *Significant p-value <0.05 CLABSI, central line-associated bloodstream infections; CAUTI, catheter-associated urinary tract infections; BSI, bloodstream infections; SSI, surgical site infection; UTI, urinary tract infection

				CLABSI				CAUTI				BSI			
		Yes	Percent	No		Yes		No		Yes		No		Yes	
				Count	Column N %	Count	Column N %	Count	Column N %	Count	Column N %	Count	Column N %	Count	Column N %
Fever+ >37.8	Yes	186	58.9%	130	53.7%	56	75.7%	126	64.9%	60	49.2%	146	59.3%	40	57.1%
	P-value			0.001*				0.006*				0.741			
UTI	Yes	130	40.9%	130	53.3%	0	0.0%	8	4.1%	122	98.4%	130	52.4%	0	0.0%
	P-value			0.000*				0.000*				0.000*			
Positive urine culture	Yes	118	37.1%	118	48.4%	0	0.0%	6	3.1%	112	90.3%	118	47.6%	0	0.0%
	P-value			0.000*				0.000*				0.000*			
Acute dysuria	Yes	16	5.0%	16	6.6%	0	0.0%	4	2.1%	12	9.7%	16	6.5%	0	0.0%
	P-value			0.024*				0.002*				0.029*			
Acute pain, swelling, or tenderness of the testes, epididymis, or prostate	Yes	22	6.9%	22	9.0%	0	0.0%	4	2.1%	18	14.5%	22	8.9%	0	0.0%
	P-value			0.007*				0.000*				0.010*			
Costovertebral angle pain or tenderness	Yes	16	5.0%	16	6.6%	0	0.0%	2	1.0%	14	11.3%	16	6.5%	0	0.0%
	P-value			0.024*				0.000*				0.029*			
Incontinence	Yes	6	1.9%	6	2.5%	0	0.0%	2	1.0%	4	3.2%	6	2.4%	0	0.0%
	P-value			0.173				0.161				0.189			
Urinary urgency	Yes	4	1.3%	4	1.6%	0	0.0%	2	1.0%	2	1.6%	4	1.6%	0	0.0%
	P-value			0.268				0.650				0.285			
Urinary frequency	Yes	4	1.3%	4	1.6%	0	0.0%	2	1.0%	2	1.6%	4	1.6%	0	0.0%
	P-value			0.258				0.650				0.285			
Suprapubic tenderness	Yes	44	13.9%	44	18.2%	0	0.0%	4	2.1%	40	32.3%	44	17.9%	0	0.0%
	P-value			0.000*				0.000*				0.000*			
Visible (gross) hematuria	Yes	28	8.8%	28	11.5%	0	0.0%	2	1.0%	26	21.0%	28	11.3%	0	0.0%
	P-value			0.002*				0.000*				0.003*			
Leukocytosis	Yes	110	34.6%	108	44.3%	2	2.7%	8	4.1%	102	82.3%	110	44.4%	0	0.0%
	P-value			0.000*				0.000*				0.000*			
Bloodstream infection	Yes	146	45.9%	72	29.5%	74	100.0%	146	75.3%	0	0.0%	76	30.6%	70	100.0%
	P-value			0.000*				0.000*				0.000*			
Chills/rigors	Yes	98	30.8%	36	14.8%	62	83.8%	98	50.5%	0	0.0%	66	26.6%	32	45.7%
	P-value			0.000*				0.000*				0.002*			
Hypotension	Yes	90	28.3%	28	11.5%	62	83.8%	90	46.4%	0	0.0%	64	25.8%	26	37.1%
	P-value			0.000*				0.000*				0.063			
At least one positive blood culture not related to contamination	Yes	146	46.2%	72	29.8%	74	100.0%	144	74.2%	2	1.6%	78	31.7%	68	97.1%
	P-value			0.000*				0.000*				0.000*			
SSI	Yes	24	7.5%	24	9.8%	0	0.0%	24	12.4%	0	0.0%	20	8.1%	4	5.7%
	P-value			0.005*				0.000*				0.511			
Purulent drainage from the superficial incision	Yes	28	8.8%	28	11.5%	0	0.0%	28	14.4%	0	0.0%	24	9.7%	4	5.7%
	P-value			0.002*				0.000*				0.301			
Abscess	Yes	14	4.4%	14	5.7%	0	0.0%	14	7.2%	0	0.0%	14	5.6%	0	0.0%
	P-value			0.035*				0.002*				0.042*			
Spreading cellulitis at the surgical site	Yes	36	11.3%	36	14.8%	0	0.0%	36	18.6%	0	0.0%	26	10.5%	10	14.3%
	P-value			0.000*				0.000*				0.375			
Healthcare-associated pneumonia	Yes	12	3.8%	12	4.9%	0	0.0%	12	6.2%	0	0.0%	12	4.8%	0	0.0%
	P-value			0.109				0.009*				0.127			
Cough	Yes	42	13.2%	28	11.5%	14	18.9%	28	14.4%	14	11.3%	40	16.1%	2	2.9%
	P-value			0.098				0.419				0.004*			
Purulent sputum	Yes	42	13.2%	30	12.3%	12	16.2%	28	14.4%	14	11.3%	38	15.3%	4	5.7%
	P-value			0.383				0.419				0.036*			
Infiltrate	Yes	56	17.6%	42	17.2%	14	18.9%	36	18.6%	20	16.1%	48	19.4%	8	11.4%
	P-value			0.736				0.579				0.124			
Prior hospitalization	Yes	172	54.1%	128	52.5%	44	59.5%	110	56.7%	62	50.0%	132	53.2%	40	57.1%
	P-value			0.290				0.242				0.561			
Transfer	Yes	52	16.4%	40	16.4%	12	16.2%	42	21.6%	10	8.1%	32	12.9%	20	28.6%
	P-value			0.971				0.001*				0.002*			
Surgery during hospitalization or in the last 12 months	Yes	190	59.7%	148	60.7%	42	56.8%	124	63.9%	66	53.2%	142	57.3%	48	68.6%
	P-value			0.549				0.058				0.088			
Prior pharmacological and/or nonpharmacological therapy	Yes	240	79.5%	176	76.5%	64	88.9%	148	82.2%	92	75.4%	196	81.0%	44	73.3%
	P-value			0.023*				0.150				0.189			
Preoperative prophylaxis	Yes	200	63.3%	154	63.6%	46	62.2%	130	67.0%	70	57.4%	154	62.6%	46	65.7%
	P-value			0.818				0.084				0.634			

Among the 318 participants, the majority (n=268, 84.3%) had not been transferred from another site. For those transferred, transfers included 4.4% (n=14) from the High Dependency Unit (HDU), 3.1% (n=10) from the Intensive Care Unit (ICU), and an equal 3.1% (n=10) from other hospitals. Smaller percentages were transferred from medical units (n=6, 1.9%), Coronary Care Unit (CCU) (n=2, 0.6%), male surgical units (n=2, 0.6%), psychiatry (n=2, 0.6%), and MS2 (n=4, 1.3%). Invasive procedures were common, with 63.2% (n=201) of patients having an intravenous (IV) line. Mechanical ventilation was used in 11.0% (n=35) of cases, and 25.8% (n=82) had a urinary catheter. Regarding patient outcomes, 186 (58.5%) remained in the hospital, while 56 (17.6%) were transferred to other departments. A total of 3.8% (n=12) were discharged, and 20.1% (n=64) of the patients unfortunately died (Table 5).

**Table 5:** Transfer sites, invasive procedures, and patient outcomes HDU, High Dependency Unit; ICU, Intensive Care Unit; CCU, Coronary Care Unit; IV, intravenous

		Count	Column N%
Site of transfer	No	268	84.3%
	Medical	6	1.9%
	HDU	14	4.4%
	ICU	10	3.1%
	Other hospital	10	3.1%
	CCU	2	0.6%
	Male surgical	2	0.6%
	Psych	2	0.6%
	Transferred to MS2	4	1.3%
Invasive procedures	IV line	201	63.2%
	Mechanical ventilator	35	11.0%
	Urinary catheter	82	25.8%
Outcome	Still in hospital	186	58.5%
	Transferred to another department	56	17.6%
	Discharged	12	3.8%
	Dead	64	20.1%

The binary logistic regression analysis provides insights into how various factors affect the risk of the three most common HAIs. Patients with three or more comorbidities are at significantly higher risk of CLABSI (OR=3.5, 95% CI: 1.46-8.36), indicating a strong association between multiple health issues and the likelihood of developing this infection. However, having three or more comorbidities did not significantly affect the risk of CAUTI (OR=0.65, 95% CI: 0.32-1.32) or BSI (OR=0.59, 95% CI: 0.26-1.32). Fever above 37.8°C was a strong indicator of increased risk for CLABSI (OR=2.68, 95% CI: 1.48-4.82) but was associated with a reduced risk of CAUTI (OR=0.52, 95% CI: 0.33-0.83). 

The study found that patients with UTIs had a reduced risk of CLABSI and BSI but a high risk of CAUTI. A positive urine culture was associated with a lower risk of CLABSI and BSI but an increased risk of CAUTI. Acute pain, swelling, or tenderness of the testes, epididymis, or prostate was a significant risk factor for CAUTI. Suprapubic tenderness was associated with a lower risk of CLABSI and BSI but an increased risk of CAUTI. Visible hematuria reduced the risk of CLABSI and BSI but significantly raised the risk of CAUTI. Leukocytosis was associated with a lower risk of CLABSI and BSI but a high risk for CAUTI. Patients with a bloodstream infection were at high risk for CLABSI and BSI but significantly reduced the risk of CAUTI. Chills or rigors increase the risk of CLABSI and BSI while reducing the risk of CAUTI. Hypotension showed a similar pattern, indicating a high risk for CLABSI but a reduced risk for CAUTI and an increased risk for BSI (Table 6).

**Table 6:** Regression analysis of the relation between the three most common HAIs and the demographic and clinical characteristics of the patients *significant at p-value <0.05 SSI, surgical site infection; UTI, urinary tract infection; OR, odd ratio

	CLABSI	CAUTI	BSI
	OR (95 %CI)	OR (95 %CI)	OR (95 %CI)
Comorbidities (3 or more)	3.5 (1.46-8.36)*	0.65 (0.32-1.32)	0.59 (0.26-1.32)
Comorbidities (2 comorbidities)	1.9 (0.78-4.59)	0.97 (0.51-1.86)	0.81 (0.38-1.69)
Comorbidities (one)	1.86 (0.73-4.70)	1.67 (0.84-3.3)	0.92 (0.42-2.01)
Smoker (former smoker)	1.66 (0.66-4.08)	0.47 (0.18-1.24)	1.28 (0.48-3.4)
Smoker (active smoker)	0.79 (0.37-1.68)	0.75 (0.40-1.41)	1.41 (0.71-2.81)
BMI (high)	1.01 (0.54-1.9)	1.1 (0.64-1.87)	0.76 (0.38-1.53)
BMI (under)	0.97 (0.51-1.86)	0.59 (0.33-1.06)	1.68 (0.90-3.14)
Physical mobility (with assistance)	1.51 (0.61-3.75)	1.00 (0.47-2.2)	0.533 (0.21-1.36)
Physical mobility (bedridden)	1.3 (0.54-2.92)	0.76 (0.38-1.50)	1.06 (0.48-2.33)
Fever+ >37.8	2.68 (1.48-4.82)*	0.52 (0.33-0.83)*	0.91 (0.53-1.56)
UTI	0.006 (0.00-0.096)*	1418.3 (296.2-6791.5)*	0.006 (0.00-0.11)*
Positive urine culture	0.007 (0.00-0.117)*	292.4 (106.7-800.9)	0.001 (0.00-0.13)*
Acute pain, swelling, or tenderness of the testes, epididymis, or prostate	0.06 (0.00-1.10)	8.06 (2.66-24.45)*	0.07 (0.00-1.19)
Suprapubic tenderness	0.03 (0.00-0.49)*	22.4 (7.75-64.56)*	0.03 (0.00-0.53)*
Visible (gross) hematuria	0.05 (0.00-0.85)*	25.5 (5.92-109.53)*	0.05 (0.00-0.91)*
Leukocytosis	0.035 (0.01-0.14)*	107.8 (46.3-250.8)*	0.008 (0.00-0.14)*
Bloodstream infection	354.5 (21.6-5798.7)*	0.00 (0.00-0.02)*	317 (19.44-5200)*
Chills/rigors	29.85 (14.6-60.85)*	0.003 (0.00-0.06)*	2.32 (1.34-4.01)*
Hypotension	39.86 (19.15-82.94)*	0.005 (0.00-0.07)*	1.69 (0.96-2.98)
At least one positive blood culture not related to contamination	350.4 (21.5-5731.8)*	0.006 (0.00-0.02)*	73.23 (17.5-307)*
SSI	0.06 (0.00-1.00)*	0.03 (0.00-0.46)*	0.69 (0.22-2.09)
Purulent drainage from the superficial incision	0.05 (0.00-0.85)*	0.02 (0.00-0.38)*	0.56 (0.18-1.68)
Abscess	0.11 (0.01-1.81)	0.05 (0.00-0.85)*	0.11 (0.00-1.94)
Spreading cellulitis at the surgical site	0.04 (0.00-0.63)*	0.02 (0.00-0.28)*	1.42 (0.65-3.11)

## Discussion

The present study aimed to estimate the prevalence and determinants of HAIs at the KAASH in Taif, Saudi Arabia. The study provided comprehensive insights into the demographic and clinical characteristics of the patient population, the types of HAIs developed, the distribution of causative organisms, and various clinical symptoms and factors associated with HAIs.

The demographic data revealed a balanced gender distribution with a slight female predominance (51.6%). The mean age of the participants was 56.44 years, which indicates a mature patient population, often more susceptible to infections due to age-related comorbidities and weakened immune systems [13-15]. Most patients were residents of Taif (76.1%), reflecting the hospital's primary service area, with smaller proportions from surrounding regions.

Occupation and educational data indicated that a significant portion of the patients (30.5%) were not working or had missing information, which may correlate with the high prevalence of chronic illnesses that prevent employment. Housewives constituted 19.5% and students 13.6%, highlighting the diverse socioeconomic background of the patients. The marital status distribution showed that 64.8% of patients were married, suggesting potential family support, which is crucial in patient care and recovery.

The comorbidity profile of the patients was extensive, with hypertension (50.9%) and diabetes (39.6%) being the most prevalent conditions. These comorbidities are known risk factors for HAIs due to their impact on the immune system and overall health status [16,17]. The study also noted significant incidences of CVA (23.9%), renal impairment (5.7%), and other chronic conditions, emphasizing the complexity of HAIs with multimorbidity [18].

Regarding the reasons for hospital admission, pneumonia was the leading cause (26.8%), followed by trauma (17.4%) and cardiovascular disease (CVD) (16.4%). These findings align with global trends where respiratory and cardiovascular conditions are major contributors to hospital admissions, especially among older adults [19,20]. Sepsis (13.9%) and diabetes mellitus (12%) were also notable causes, underlining the importance of infection control and management of chronic diseases in reducing hospital admissions and aligning with previous research [21]. The types of HAIs developed among the patients were predominantly CAUTI (39%) and CLABSI (23.3%). These findings are consistent with global data, where CAUTI and CLABSI are among the most common HAIs in hospital settings [22-25]. The presence of ventilator-associated pneumonia (VAP) and decubitus ulcers further indicates the need for continuous monitoring and preventive measures in critical care units as findings of previous research indicated their effectiveness in reducing and managing HAIs in critical care units [26].

The causative organisms of HAIs were diverse, with *Klebsiella pneumoniae *being the most common (30.2%), and *Acinetobacter baumannii *and* Escherichia coli *each accounted for 10.1% of infections, which is similar to the results of previous studies [27-29]. The presence of multiple resistant organisms, such as MRSA (5%), highlights the challenges in managing HAIs and the importance of antimicrobial stewardship programs that have been proven to be effective [30]. MRSA is a leading cause of HAIs, especially in acute care institutions, where it can result in serious consequences such as BSI and pneumonia. The emergence of antibiotic resistance, notably in MRSA, is associated with antibiotic overuse and misuse. Antibiotic prescriptions have been linked to an increase in HA-MRSA infections, and higher outpatient antibiotic use has reported higher rates of HA-MRSA, implying that minimizing unnecessary antibiotic prescriptions could be a critical strategy for controlling these infections [30,31].

Clinical symptoms associated with HAIs were varied, with fever (>37.8°C) being reported in 58.9% of cases. UTIs were confirmed in 40.9% of patients, with positive urine cultures in 37.1%. Acute dysuria and other specific symptoms were less common, indicating the variable presentation of HAIs. BSI were noted in 45.9% of patients, with significant incidences of chills, rigors, and hypotension, suggesting severe systemic responses to infections. This variable presentation of HAIs is a leading cause of challenges associated with HAI management, requiring more resources [18]. Patient outcomes showed that 58.5% of the patients remained hospitalized, while 17.6% were transferred to other departments. The discharge rate was low at 3.8%, and the mortality rate was notably high at 20.1%. These outcomes reflect the severe nature of the HAIs, the possible effect of antibiotic resistance, and the underlying health conditions of the patients that influence their outcomes [32]. The high mortality rate underscores the critical need for effective infection control measures and comprehensive care strategies to improve patient outcomes. Enhanced infection control practices, such as stringent hand hygiene, proper sterilization procedures, and effective isolation protocols, are essential to curb the spread of infections. Additionally, improving staff training and awareness regarding infection prevention protocols can significantly mitigate HAI risks.

Invasive procedures were common, and the high prevalence of invasive procedures correlates with the risk of developing HAIs, confirming previous research findings and emphasizing the need for stringent aseptic techniques and continuous monitoring [14]. The regression analysis showed that significant predictors of CLABSI included weight, physical mobility, fever >37.8, smoking status, hypotension, organism(s) identified in blood not related to an infection at another site, surgery during hospitalization or in the last 12 months, invasive procedures, and preoperative prophylaxis. These factors align with previous studies in Middle Eastern countries and elsewhere that emphasize the role of a patient's weight, mobility, and procedural history in infection risk [33,34].

The significant association between having three or more comorbidities and a higher risk of CLABSI (OR=3.5) aligns with established literature, which suggests that patients with multiple morbidities have weak immune responses and are more susceptible to infections. For instance, studies by Alhumaid et al. and Kaye et al. emphasized that patients with chronic conditions, particularly those requiring invasive procedures like central lines, are more vulnerable to CLABSI [35,36]. However, the lack of a significant association between multiple comorbidities and the risk of CAUTI and BSI in this study contrasts with the findings of other studies that reported that comorbidities like diabetes and renal impairment increased the risk of CAUTI [37,38]. This discrepancy could be attributed to the differing patient populations, settings, or the types of comorbidities studied. It may also suggest that specific comorbidities rather than the number of comorbidities might play a more critical role in the development of CAUTI and BSI.

The association between fever above 37.8°C and an increased risk of CLABSI (OR=2.68) is consistent with prior research. Fever has long been recognized as a common symptom of BSI, including CLABSI [36]. However, the observation that fever is associated with a reduced risk of CAUTI (OR=0.52) introduces a novel perspective. This might be explained by the fact that fever in hospitalized patients may prompt more aggressive diagnostic and treatment interventions, which could inadvertently reduce the risk of developing CAUTI. This finding contrasts with previous evidence where fever was a frequent symptom of CAUTI, particularly in patients with more severe infections [39-41].

The finding that patients with UTIs had a reduced risk of CLABSI and BSI but a high risk of CAUTI aligns with the literature on the natural history of these infections. UTIs are the most common cause of CAUTI, and having a UTI increases the risk of this specific infection [39,40]. Positive urine cultures serve as strong predictors for CAUTI, but their association with lower risks of CLABSI and BSI raises interesting questions. It suggests a potential inverse relationship, where patients with UTIs may be receiving more targeted urinary care, thereby reducing the risk of central line and BSI.

The association between suprapubic tenderness and an increased risk of CAUTI but a lower risk of CLABSI and BSI is consistent with the localizing symptoms of UTIs. Similarly, visible hematuria raises the risk of CAUTI while reducing the risk of CLABSI, and BSI follows the same logic. These symptoms likely direct clinical focus toward the urinary system, resulting in enhanced prevention and treatment efforts for other types of infections. This supports existing research that localized symptoms can guide the diagnostic and treatment pathways in hospital settings due to enhanced early recognition and treatment of infections, which reduce the risk of secondary infections [42].

This study’s findings suggest that there may be an inverse relationship between the risk of different HAIs. Further research could explore whether the aggressive management of one infection reduces the likelihood of developing another, which could help tailor hospital infection control policies. Instead of focusing on the number of comorbidities, future research could explore which specific comorbidities pose the greatest risk for each type of HAI. This idea is supported by previous research, which emphasized the need for stratified risk assessments in infection prevention [43]. Despite providing valuable insights into the HAI burden at KAASH, which is vital to improving care quality and optimized outcomes, this study presents notable limitations. The study, conducted at a single hospital with a small sample size of 318 patients, may not be generalizable to other hospitals or regions due to its cross-sectional design, self-reported data, exclusion criteria, and limited scope. The cross-sectional design provides a snapshot of the situation, making it difficult to establish causality or observe changes over time. The study also excluded patients from outpatient areas, emergency departments, skilled nursing, and rehabilitation units, potentially overlooking important sources of HAIs. Additionally, the study did not consider other potential confounding variables that might influence HAIs, such as environmental cleaning and disinfection practices, healthcare providers' adherence to infection control measures like hand hygiene, and the availability of personal protective equipment. Therefore, future extensive multicenter longitudinal studies are recommended, and they should consider a broader range of HAIs, including diverse patient populations, and account for additional potential confounding variables to enhance the generalizability and robustness of the findings.

## Conclusions

The study at KAASH revealed a high burden of HAIs, with catheter-associated urinary tract and central line-associated BSI being the most common. Microbial resistance is a major issue, with hypertension and diabetes being major comorbidities. The study emphasizes the need for robust infection control measures and targeted interventions to reduce HAI incidence and improve patient outcomes. It suggests establishing surveillance systems, implementing evidence-based guidelines, and providing continuous education and training for healthcare personnel. Addressing these determinants can lead to better patient outcomes and more efficient healthcare delivery.
